# Lymphedema Impact and Prevalence International Study: The Canadian Data

**DOI:** 10.1089/lrb.2019.0014

**Published:** 2019-04-22

**Authors:** David H. Keast, Christine Moffatt, Ashrafunissa Janmohammad

**Affiliations:** ^1^ Department of Family Medicine, Schulich School of Medicine and Dentistry, Western University, London, Canada.; ^2^ Parkwood Institute Research, St. Joseph's Health Care London, Lawson Health Research Institute, London, Canada.; ^3^ School of Social Sciences, Nottingham Trent University, Nottingham, United Kingdom.

**Keywords:** Canada, prevalence, LIMPRINT, chronic edema, lymphedema, lymphoedema

## Abstract

***Background:*** Chronic edema/lymphedema is defined as edema present for more than 3 months. It is underrecognized and undertreated. The International Lymphedema Framework developed an international study, Lymphedema Impact and Prevalence International (LIMPRINT), to estimate the prevalence and impact of chronic edema in heterogeneous populations. Canada participated in this study.

***Methods and Results:*** Participants were recruited from an outpatient chronic wound management clinic. At a study visit, the following tools were administered: The Core Tool, Demographics and Disability assessment (WHODAS 2.0), Quality-of-life assessment (LYMQOL + EQ-5D), Details of swelling, Wound assessment, and Cancer. Data were entered into an international database (Clindex), and country-specific data were analyzed. Sixty-eight subjects were enrolled. Fifty-seven percent were males and 43% females. More than 90% were older than 45 years. Only 7.35% had primary lymphedema. Most had lower extremity edema (65 of 68). Over half (47.06%) were morbidly obese with body mass index of >40. The most common underlying condition was venous disease. Only 8 of 68 had a history of cancer. While 72.06% had a history of cellulitis, only 10.2% had been hospitalized in the past year. 39.71% had an open wound. More than 75% had received multilayer bandaging, compression garments, wound dressings, and extensive counseling. Few had received manual lymphatic drainage, which is not funded. Disability was less than expected.

***Conclusion:*** Chronic edema/lymphedema is an underrecognized condition. These data and the wider LIMPRINT study are important tools to advocate for wider recognition and funding of treatment by health care systems.

## Introduction

Chronic edema is defined as edema present for more than 3 months, affecting any site of the body irrespective of underlying etiology or concurrent comorbidities.^1^ Many authors now define all chronic edema as lymphedema.^2^ In the peripheral tissue, capillaries and the lymphatics are intimately associated and function as a coordinated unit to manage tissue fluid. Thus, any chronic edema will involve lymphatic dysfunction. Lymphedema has been described as a hidden epidemic. Worldwide, it is underrecognized and undertreated. It is a chronic condition and is best treated using the chronic disease models that focus on self-management with professional support for assessment, treatment recommendations, and monitoring.^3^

A recent systematic review has indicated that there is a dearth of population-based epidemiological studies to define the prevalence of chronic edema and little robust evidence of the cost and clinical effectiveness of different models of care.^4^ It is therefore difficult for those who commission services, and other health care agencies, to determine treatment priorities and to justify the provision of clinical services for people with chronic edema or to reimburse care costs. Recently, the Canadian Lymphedema Framework (www.canadalymph.ca) estimated the prevalence of lymphedema in Canada by using available data for the prevalence of conditions known to be associated with lymphedema. The estimated number of Canadians living with lymphedema is 1 million giving a prevalence of 2.8%.

## The Lymphedema Impact and Prevalence International Study

This international study was designed to estimate the prevalence and impact of chronic edema in heterogeneous populations.

### Primary aim

To determine the prevalence and functional impact of chronic edema in the adult population at a national and international level using modular epidemiological tools.

### Secondary aims

1. To identify, using a case ascertainment questionnaire, all patients within defined populations of participating countries, who currently suffer from chronic edema for more than 3 months duration and who are patients within the health care system.
2. To determine the impact of chronic edema on the lives of patients using questionnaires as follows:
  a. Demographic and disability assessment
  b. Health-related quality of life
  c. Details of swelling
  d. Wound assessment
  e. Cancer assessment
3. To estimate the proportion of patients with chronic edema who also have a wound in the same anatomical region.

### Inclusion criteria

1. The patient is older than 18 years.
2. The patient has swelling for more than 3 months.
3. The patient is able to understand the study as set out in the information sheet.
4. The patient is able to give informed consent.

### Exclusion criteria

1. The patient is unwilling or unable to participate for whatever reason.
2. The patient is receiving end-of-life care.
3. It is considered not to be in the patient's best interest as decided by the lead clinician.

### Data collection tools

The Core Tool was the case ascertainment tool. The components are shown Table 1. All sites completed the Core Tool. There was a series of Modular Tools, which were optional. These included: Demographics and Disability assessment (WHODAS 2.0), Quality-of-life assessment (LYMQOL + EQ-5D), Details of swelling, Wound assessment, and Cancer details. Only a limited number of sites completed the Modular Tools.

**Table 1.** Core Tool

*Components of Core Tool*
Type of facility in which data are collected
Level of obesity
Relevant comorbidities
Mobility
Classification of lymphedema
Lymphedema history
Cellulitis history
Categories of treatment
Site of swelling
Wound area
Access to treatment
Subjective control of swelling

Forty sites in 9 countries participated in the study. These countries included Australia, Canada, Denmark, France, Ireland, Italy, Japan, Turkey, and the United Kingdom.

## Canadian Site

### Site description

The study was conducted at the Parkwood Institute Research, St. Joseph Health Care London, which is part of Lawson Health Research Institute in London, ON, Canada. Lawson is the largest hospital-based research institute in Canada and works in partnership with the Western University. The mission is to understand the basis of wellness and the dysfunctions of the body and mind that result from disease.

Our service area includes the South West Local Health Integration Network (SWLHIN), which covers a large portion of South Western Ontario Canada, from Lake Erie to the Bruce Peninsula. This area is 21,639 km^2^, with a population of 962,539. The health region is geographically large, as it takes 5 hours to drive from top to bottom. Over half of the population lives in the Metropolitan London Census Area.

The Parkwood Chronic Wound Management was founded in 1997 and sees persons with chronic wounds of any etiology. Since 2006, the clinic has maintained an administrative database of clinic patient visits categorized by the International Classification of Disease code for the most responsible diagnosis for the visit. Lymphedema is the most responsible diagnostic code for 670 patients observed from June 2006 to July 2018. This represents 24% of our clinic volume. In 2014, a complete audit of 326 lymphedema patient charts was conducted.^5^ Our patients at that time were evenly distributed between males and females, primarily involved the lower limb and had 7.3 comorbid conditions. Forty-six percent were morbidly obese.

### Aims of the Canadian study

1. To determine the prevalence and functional impact of chronic edema in the adult population attending a chronic wound management clinic in the SWLHIN.
2. To determine the impact of chronic edema on the lives of patients by using five module tools.
3. To estimate the proportion of patients with chronic edema, who also have a wound in the same anatomical region.

## Methods

The Lymphedema Impact and Prevalence International (LIMPRINT) study was approved by the Western University Human Research Ethics Board in March 2016. A contract was signed between the International Lymphedema Framework and Lawson Health Research Institute in May 2016. Funding was obtained from the St. Joseph's Health Care Foundation. Data collection started in June 2016. Patients were recruited directly from the Chronic Wound Management Clinic. On the clinic visit days, all lymphedema patients seen between June 6, 2016, and December 11, 2017, were assessed for eligibility for inclusion into the LIMPRINT study by the principal investigator. Based on the patient's verbal approval, the letter of information was provided for them to read at home. Permission was granted by the participant for the research coordinator to contact them by telephone at least 2 days after the clinic visit to offer an appointment for a study visit to the research center. Any immediate questions were answered. On the study visit day, the letter of information was explained, and informed consent was obtained before the start of data collection.

After review of the letter of information and consent signature, the Core Tool was administered, and the following Module Tools were also administered by the Research Coordinator: Demographic and Disability Tool, Details of Swelling Tool, Wound Tool, and Quality-of-Life Tool EQ-5D. The Filariasis Tool was not administered as lymphatic filariasis is not endemic locally. Information used to complete each tool included patient report, review of clinic notes, and clinical examination. Most of the data were extracted from clinic notes in the hospital electronic medical record. Weight and height were measured in the clinic, and body mass index (BMI) was automatically calculated by the electronic medical record. Wound related data were extracted from the most recent clinic visit, which had occurred in the previous week. The EQ-5D was independently completed by the patient. Data were entered into a paper-based tool and later transcribed into the Clindex electronic database. After data entry was closed, all Clindex data were verified against the paper record.

## Results

Internationally, there were 13,016 participants in the Core Tool, of which 1359 participants completed the Module Tools. Only Canadian data were available from the Core Tool, but both Canadian and international data were available for the Module Tools. From June 2016 to December 2017, our site enrolled 68 patients in both the Core Tool and the Module Tools. Of 248 contacted, 180 (73%) patients were either unable to join or declined to join the study. Main reasons for non-enrollment were:

1. Travel distance to clinic. (Some patients drive 4 hours to get to clinic).
2. Language barrier.
3. Unable to participate due to cognitive impairment.
4. Unable to schedule Paratransit.
5. Declined to participate.

### Demographics

**FIG. 1.**
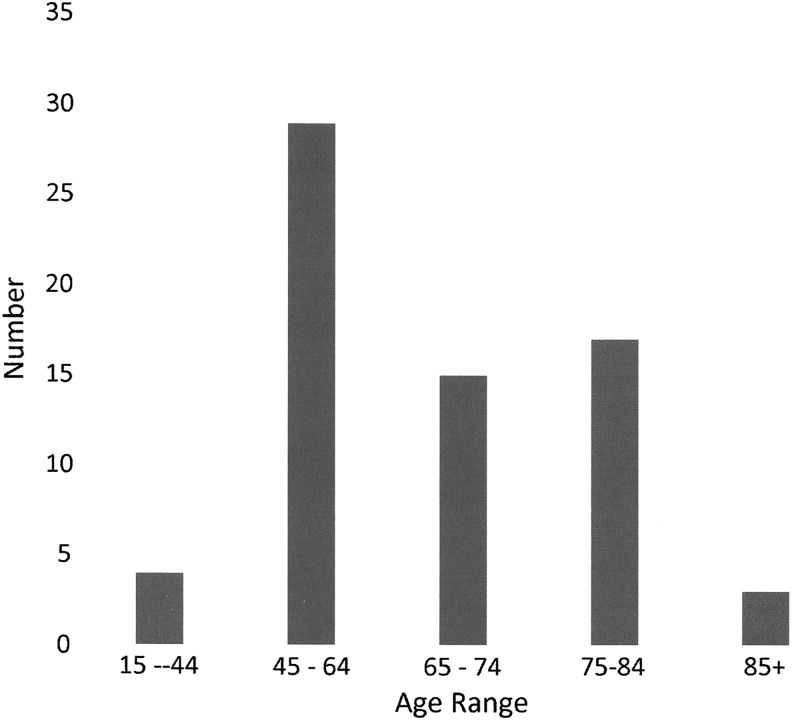
Age distribution.

**FIG. 2.**
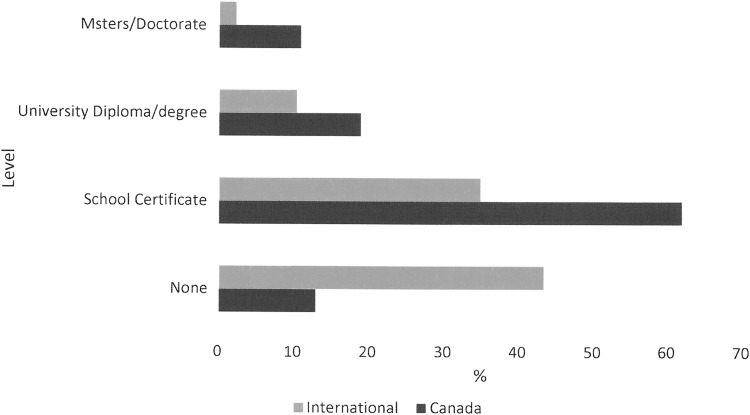
Level of education. *Note:* None indicates completion of primary education but no secondary graduation diploma.

Gender was relatively evenly balanced with 57% males and 43% females. Most participants were older than 45 years (94%). Age distribution is shown in Figure 1. Thirty-two percent lived alone, whereas 68% lived with a spouse, relative, or friend. Internationally, fewer people lived alone (14.8%). Almost two thirds (63%) were the prime financial supporter of the family, whereas internationally the participant was less likely to be the prime supporter (24.9%). Sixty-nine percent owned their home, and 84% owned a car. Internationally, there was comparable home ownership at 77.3%, but car ownership was much less at 58.7%. The participants were in general well educated as shown in Figure 2. Mean age at which Canadian participants left full-time education was 20.4 ± 7.4 years compared with 17.4 ± 5.6 internationally. Employment status is compared in Table 2.

**Table 2.** Employment Status

*Status*	*Canada (%)*	*International (%)*
Employed full time	16.2	14.3
Employed part time	32.6	3.3
Retired	57.4	28.6
Unemployed looking for work	0	2.4
Not working due to illness	11.8	6.5
Looking after the house	2.9	43.4
Full- or part-time education or training	1.5	1.3
Other	17.6	2.6

### Comorbidities

**FIG. 3.**
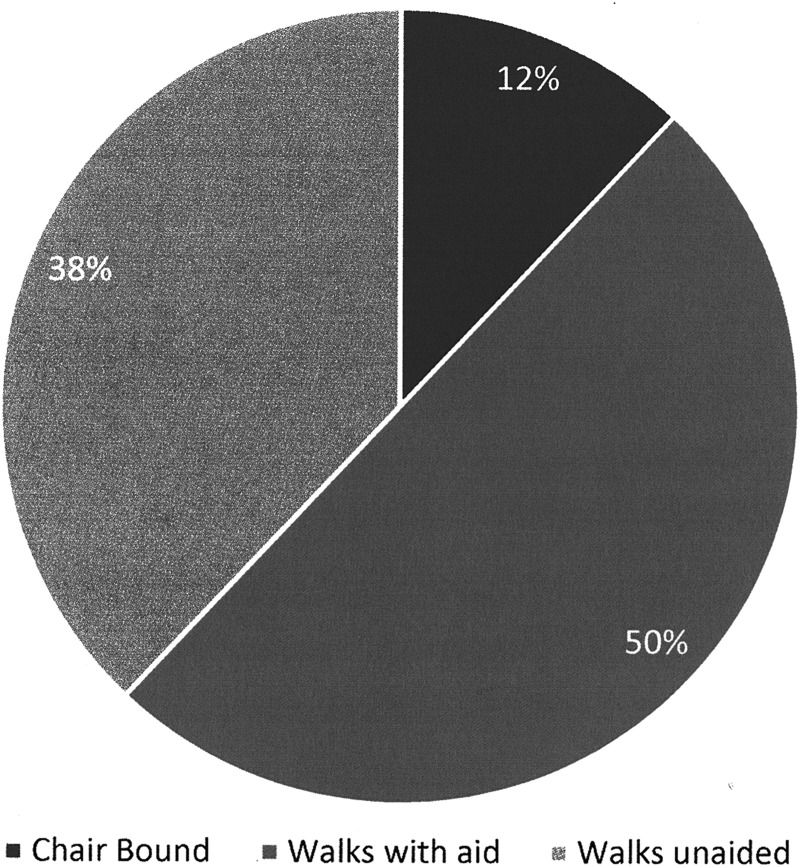
Mobility.

In our chart audit from 2014,^5^ we found that our patients had a mean of 7.3 comorbidities. In this study, 39.1% were found to have diabetes (38% in the audit), 20.59% with congestive heart failure and/or ischemic heart disease, 11.76% with neurological disorders, and only 2.94% with peripheral artery disease. Morbid obesity (BMI of >40) accounted for 47.06% of participants, which was consistent with 45% in the chart audit. Very few participants had problems with upper limb mobility (4%), but lower limb mobility issues affected a significant number of participants, as shown in Figure 3.

### Lymphedema characteristics

**FIG. 4.**
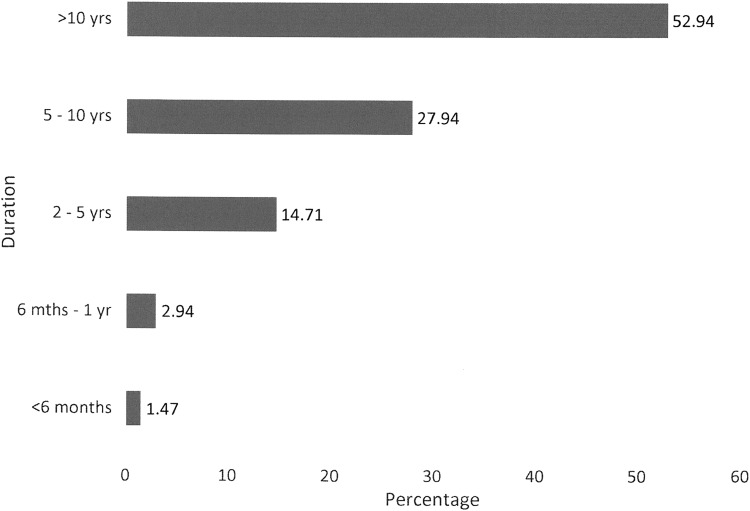
Duration of lymphedema.

Only 7.35% were deemed to have primary lymphedema, with secondary lymphedema accounting for 92.65% of cases. Secondary causes are shown in Table 3. Most participants had lymphedema for more than 5 years (Fig. 4). While 72.06% reported at least on episode of cellulitis in the past, only 10.9% reported hospitalization in the past year. Only two people reported more than one hospitalization.

**Table 3.** Causes of Secondary Lymphedema

*Condition*	*%*
Venous disease	72.06
Obesity	54.41
Noncancer other	14.71
Cancer	8.82
Treatment-related obstruction	8.82
Metastatic disease	8.82

*Note:* Percentages do not add to 100% as participants had more than one cause.

### Location and details of swelling

**FIG. 5.**
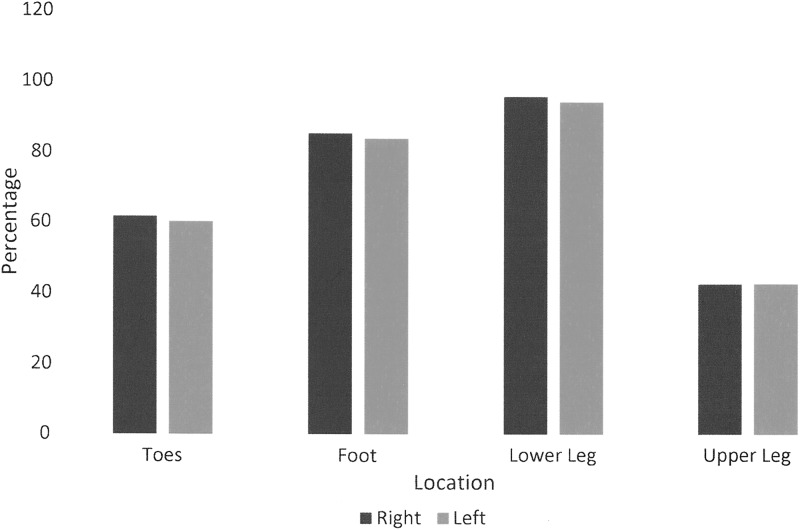
Comparison of location of lower extremity edema.

No participants had swelling of the head and neck or genitals. Three patients had swelling of the left arm, but only two had swelling extend to the fingers. Three participants had swelling of the right arm, but the upper arm was involved in only one. Most participants reported no swelling in the midline or trunk. Four participants reported buttock swelling. The majority of patients seen in our center had multifactorial lower extremity chronic edema. Location of lower limb swelling is shown in Figure 5. Comparison between Canadian and international swelling details data is shown in Table 4.

**Table 4.** Details of Swelling

*Variable*	*Canada (%)*	*International (%)*
Pitting		
Yes	67.7	58.1
No	32.4	41.9
Tissue		
Soft	79.4	71.5
Hard	20.6	28.5
Shape distortion		
Yes	26.5	35.2
No	73.5	64.8
Has patient been told reason		
Yes	77.9	62.2
No	22.1	37.8
Limb involved		
Arm only	0	37.4
Leg	95.6	59.2
Both	4.4	3.4
Stemmers sign positive		
Right hand	66.7	21.5
Left hand	66.7	22.4
Right foot	80.7	47.2
Left foot	83.8	48.7
Severity		
ISL stage I	11.8	27.9
ISL stage II	66.2	58.7
ISL stage III	22.1	13.4

ISL, International Society of Lymphology.

### Lymphedema and cancer

The Canadian site sees few cancer patients as these people are followed primarily in cancer treatment centers. For comparison with international and Canadian data, see Tables 5 and 6.

**Table 5.** Cancer Type

	*Canada (*n* = 8),* n *(%)*	*International (*n* = 816),* n *(%)*
Bladder cancer	1 (12.5)	3 (0.4)
Breast cancer	1 (12.5)	633 (77.6)
Cervical cancer	2 (25.0)	42 (5.1)
Colorectal cancer	1 (12.5)	5 (0.6)
Endometrial cancer	0	77 (9.4)
Head and neck cancer	1 (12.5)	2 (0.2)
Melanoma cancer	1 (12.5)	7 (0.9)
Ovarian cancer	0	29 (3.6)
Vulval cancer	0	2 (0.2)

**Table 6.** Time to Onset of Swelling After Cancer Treatment

	*Canada (*n* = 8/68),* n *(%)*	*International (*n* = 816/1359),* n *(%)*
<3 months	3 (37.5)	160 (19.7)
3–11 months	0	261 (32.1)
1–5 years	3 (37.5)	279 (34.4)
6–9 years	1 (12.5)	44 (5.4)
10+ years	1 (12.5)	50 (6.2)
Unknown	0	10 (1.2)
Not applicable		8 (1.0)

### Treatments

Treatments received are detailed in Table 7.

**Table 7.** Treatment Details

*Treatment*	*%*
Skin care advice	97.06
Wound dressings	95.59
Antibiotics	85.29
Massage	1.47
Physiotherapy	1.47
Compression garments	76.47
Multilayer bandaging	85.29
Pneumatic compression pumps	1.47
Debulking lipedema surgery	0
Exercise advice	79.41
Cellulitis advice	76.47
Psychological support	1.47
Other complex decongestive therapy	1.47

### Wounds and lymphedema

**FIG. 6.**
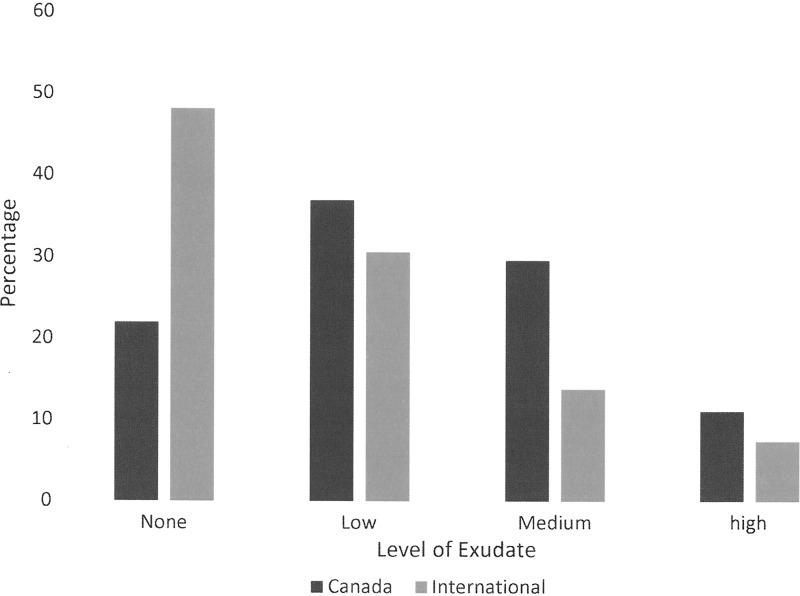
Comparison of exudate levels.

**FIG. 7.**
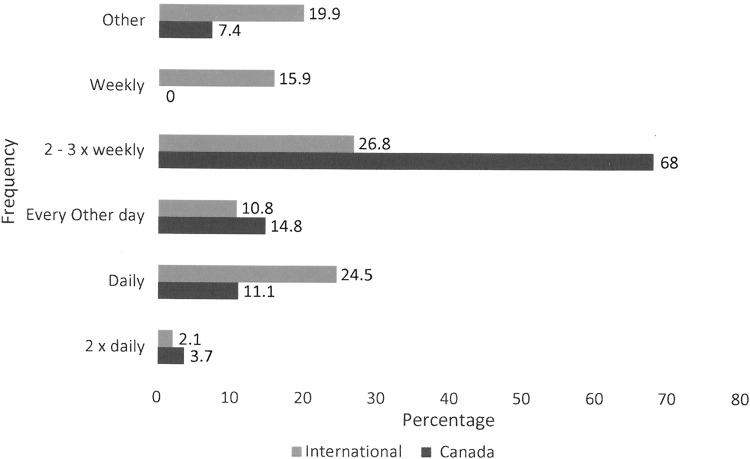
Dressing change frequency.

In this study, 39.71% of the Canadian participants had a wound, which is compared with 64% found in the 2014 chart. Nurses in both Canada and internationally are the primary wound care providers, with more than 85% indicating that a nurse was involved in their care. In Canada, all nursing care was delivered in the community, whereas internationally most nurses were hospital based. Self-care and informal caregivers were involved in one third of cases. Three quarters had three or fewer wounds. In Canada, only one wound was reported as a pressure ulcer compared with 13% internationally. There were no surgical wounds reported from Canada, whereas internationally 67.6% of 651 wounds were reported as surgical, but few of these were open and most had been closed primarily. In Canada, 26 of 27 wounds were lower extremity and 84.6% were considered venous. Exudate levels are compared in Figure 6. Despite that exudate levels were higher in Canada, only 47.6 considered the level problematic as opposed to 79% internationally. Wound size in Canada was much smaller with 85.2% being <10 cm^2^ compared with 18.4% internationally. Possible infection rates were more than double at 29.6% in Canada as opposed to 11.3% internationally. Wound durations were different. In Canada, almost 60% had a wound duration >6 months, whereas only 25.4% in the international data set had a duration >6 months. Dressing change frequencies are compared in Figure 7.

### Access to care

In our Local Health Integration Network, all treatments are fully funded for all patients except manual lymphatic drainage (MLD). Limited funding is available for physiotherapy through home and community care primarily for exercise programs. Pneumatic compression pumps are funded only for primary lymphedema. Despite this, 71.79% of males and 51.72% of females responded that their entire care was covered. This gender difference reached statistical significance at *p* = 0.0895. Few participants indicated that travel distance was a barrier to specialist care (males = 5.13%, females = 6.90%). Only one male and one female indicated that lack of transport was barrier. No patients were hospitalized. Only one female reported that her lymphedema prevented her from being discharged from home and community care.

### Impact of lymphedema

A relatively low percentage of Canadian participants, 20.6%, responded that their lymphedema affected their work life leading to a change of job or educational activities. The same percentage reported reduced income. More than 75% continued to work. Those who stopped work had done so a mean of 12.5 years previously. 26.8% reported that family income was adversely affected by their swelling. Results of the WHODAS 2.0 showed that the most adversely affected domains were standing for more than 30 minutes, 48.5% severe/extreme, and walking long distance such as 1 km, 75% severe/extreme. Difficulties were reported as present for an average of 18.3 ± 13.1 days out of 30. On average out of 30 days, participants cut back on activities for 9.0 ± 10.8 days and were unable to carry out normal activities for 5.8 ± 9.2 days. The overall WHODAS score for the group was a mean of 32.8 ± 22.2, where 0 is no disability and 100 is maximum.

**FIG. 8.**
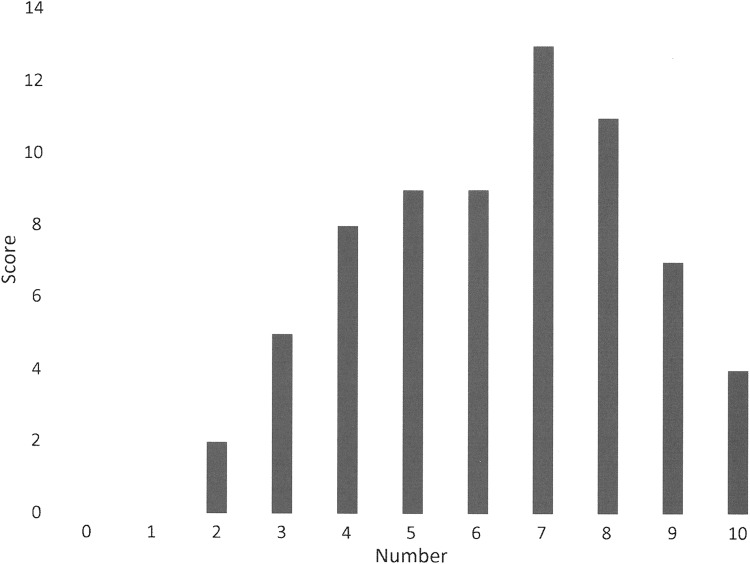
Overall quality-of-life score.

The EQ-5D detailed scores are shown in Table 8. Overall, the participants rated their quality of life at mean of 0.51 ± 0.33, where 1.0 is the best quality of life. When asked to use a visual analogue scale to rate their quality of life on that day, the mean score was 61.6 ± 19.9, where 100 is the best quality of life score. The international scores were similar at 0.57 ± 0.33 and 61.8 ± 20.6. The final quality-of-life tool used was the LYMQOL. The LYMQOL overall scores are shown in Figure 8. Canadian data are mostly related to the lower extremity. Individual domains most affected were mobility and some activities of daily living. Least affected were the emotional domains with ∼80% reporting little or no effect. International data are not compared as it related mostly to the upper extremity.

**Table 8.** EQ-5D Scores

*Domain*	*Question*	n *(%)*
Mobility	I have no problems in walking about	16 (23.9)
	I have some problems walking about	51 (76.1)
	I am confined to a bed	0
	Missing	1
Self-care	I have no problems with self-care	31 (45.6)
	I have some problems washing or dressing myself	34 (50.0)
	I am unable to wash or dress myself	3 (4.4)
	Missing	0
Usual activities	I have no problems with performing my usual activities	20 (29.4)
	I have some problems with performing my usual activities	42 (61.8)
	I am unable to perform my usual activities	6 (8.8)
	Missing	0
Pain/discomfort	I have no pain or discomfort	13 (19.1)
	I have some moderate pain or discomfort	43 (63.2)
	I have extreme pain or discomfort	12 (17.7)
	Missing	0
Anxiety/depression	I am not anxious or depressed	36 (52.9)
	I am moderately anxious or depressed	27 (39.7)
	I am extremely anxious or depressed	5 (7.4)
	Missing	0

## Discussion

This study gives an estimate of the prevalence of lymphedema in the defined population of adults attending a chronic wound management clinic, which is a specialist referral center for a population of just under 1 million people. No other center provides a comprehensive assessment and diagnosis. The clinic provides recommendations for care, education, and monitoring of care. At the time of the study, lymphedema patients represented 24% of the clinic volume. The study results do provide a clear picture of the characteristics of these patients and the impact lymphedema has on their lives. The small sample size and the large number of patients who were unable to participate is a weakness of the study, but the results are completely consistent with a previous chart audit conducted in 2014 of 326 patients.^5^ This suggests that it is a representative sample. It is unusual to see a higher proportion of males to females in published prevalence data. This relates to the location of the lymphedema primarily in the lower limbs in this patient population. The involvement of the lower limb also accounts for the significant percentage, 62%, of mobility issues, as shown in Table 5. The causes of secondary lymphedema in this study as shown in Table 6 are consistent with chart audit, 72% versus 73% venous. Table 7 shows that over half of the participants had their lymphedema for more than 10 years. Many participants had chronic swelling for many years before receiving a diagnosis. Table 9 shows that the difference between the Canadian and international data is explained by the predominance of lower leg edema in the Canadian participants. This also demonstrates that lower limb lymphedema is associated with more advanced stages of lymphedema, 88.3% Stage II or greater as opposed to 72.1%. Table 5 clearly demonstrates that the international data are heavily skewed toward breast cancer survivors and hence the emphasis on arm morbidity. Caution in interpreting these data is recommended given that they represent the smaller subset of participants who completed the Module Tools. In this study, 39.71% of participants had an open wound compared with 64% in the chart audit. The difference relates to the time frame. In the study, it is the percentage with a wound at the study visit, whereas in the audit, it was any history of a wound. Again, the location, duration, and severity of wounds are related to upper versus lower extremity location. Lower extremity wounds are generally more complex and are associated with many more comorbid conditions especially edema causing conditions, such as congestive heart failure, liver failure, and renal failure. They are far more prone to infection. Location of provision of care was different and related to international data being heavily weighted toward post breast cancer surgery. Care was predominantly hospital based. In Canada, it was based in the community and is fully funded by the provincial health care system. The difference in dressing change frequency, shown in Figure 7, relates to standard of care for dressing changes being two to three times weekly in home care. The statistically significant difference between males and females in whether their care was fully funded was surprising given that all care is funded except for MLD and intermittent pneumatic compression pumps. It may relate to females having greater expectation that the MLD should be covered.

**Table 9.** Comparison of Two Canadian Lymphedema Clinic Chart Audits

*Characteristic*	*Shallwani et al.^7^*	*Wang and Keast^5^*
Mean age, years	61.4	At diagnosis = 66.8
Female	85%	52.1%
Male	15%	47.9%
Cancer	81%	10%
Primary	7%	4%
Secondary	93%	96%
Upper body	51%	1.2%
Lower body	45%	99.7%
Unilateral	74%	17%
Bilateral	25%	83%

The overall WHODAS score is relatively low at 32.8 given that the normative value for ages 55–64 years is 3.4 ± 5.7.^6^ It is not surprising that most disability was related to standing and walking since 95.6% had lower lymphedema. The EQ-5D overall score of 0.51 ± 0.33 is somewhat lower than that obtained from the visual analogue scale of 61.1 ± 19.9 but is within the standard deviation. The EQ-5D does not ask specifically about lymphedema but is an overall quality-of-life score and can be affected by other comorbid conditions. The LYMQOL, however, does ask specifically about quality of life as it relates to living with lymphedema. When the scores are averaged the mean comes to 6.2, which is quite consistent with the EQ-5D scores.

Interestingly, the comparison between international and Canadian data reflects that between two Canadian clinics. We have previously discussed our data.^5^ In 2017, Shallwani et al.^7^ reported on an audit of 429 patients observed in their lymphedema clinic in Montreal in the previous 2 years. The comparison is shown in Table 9. Not unlike the international data, Dr. Towers sees predominantly female patients with breast cancer-related arm morbidity. It is predominantly unilateral. Lower limb lymphedema patients tend to be relatively evenly divided between males and females, to have bilateral involvement and to have more complex chronic edema. We are seeing more patients with lymphedema post hip and knee replacements. With the aging population and growing rates of obesity in developed countries, we likely to see increasing rates of lower extremity lymphedema.

## Conclusion

This study reports on the characteristics of a defined population of lymphedema patients being treated in an outpatient chronic wound management clinic. These patients have balanced gender representation and have predominantly lower limb lymphedema, which is usually bilateral and complex. Over one third have a concurrent lower extremity wound and they are prone to cellulitis. While upper limb lymphedema after breast cancer surgery has received widespread attention, lower extremity lymphedema is in general poorly recognized. With increasing risk factors such advancing age, decreased mobility, longstanding venous disease, hip and knee replacements, and growing rates of obesity, this problem is likely to become more prevalent. Early recognition and treatment will become important to reduce morbidity. Data such as this and the wider LIMPRINT study are important tools to advocate for wider recognition and funding of treatment by health care systems.
